# Structural biology of voltage-gated calcium channels

**DOI:** 10.1080/19336950.2023.2290807

**Published:** 2023-12-07

**Authors:** Xia Yao, Shuai Gao, Nieng Yan

**Affiliations:** a TaiKang Center for Life and Medical Sciences, School of Pharmaceutical Sciences, Wuhan University, Wuhan, China; b Beijing Frontier Research Center for Biological Structures, State Key Laboratory of Membrane Biology, Tsinghua-Peking Joint Center for Life Sciences, School of Life Sciences, Tsinghua University, Beijing, China; c Shenzhen Medical Academy of Research and Translation, Shenzhen, China

**Keywords:** Voltage-gated calcium channels, cryo-EM, working mechanism, structural pharmacology, drug discovery

## Abstract

Voltage-gated calcium (Ca_v_) channels mediate Ca^2+^ influx in response to membrane depolarization, playing critical roles in diverse physiological processes. Dysfunction or aberrant regulation of Ca_v_ channels can lead to life-threatening consequences. Ca_v_-targeting drugs have been clinically used to treat cardiovascular and neuronal disorders for several decades. This review aims to provide an account of recent developments in the structural dissection of Ca_v_ channels. High-resolution structures have significantly advanced our understanding of the working and disease mechanisms of Ca_v_ channels, shed light on the molecular basis for their modulation, and elucidated the modes of actions (MOAs) of representative drugs and toxins. The progress in structural studies of Ca_v_ channels lays the foundation for future drug discovery efforts targeting Ca_v_ channelopathies.

## Introduction

**Figure 1.**
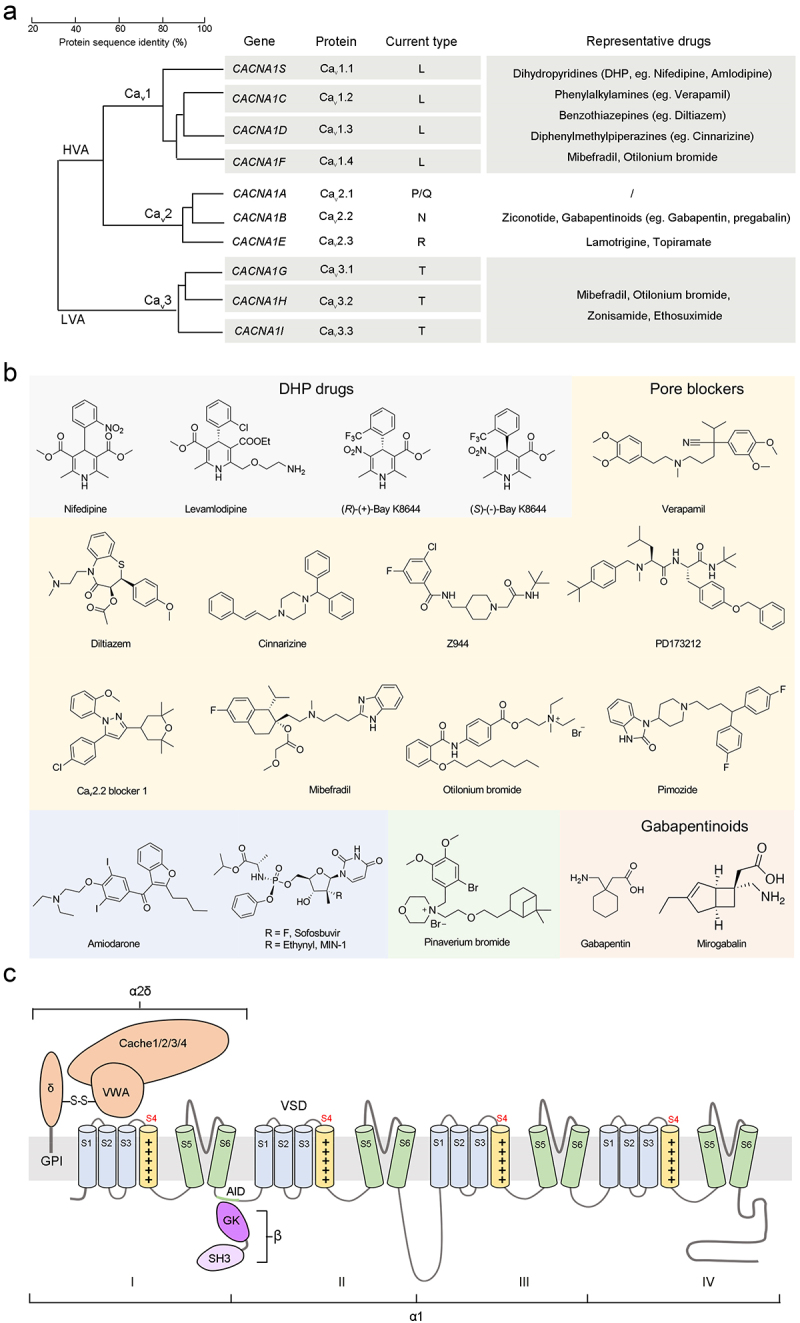
Classification, pharmacology, and topology of Ca_v_ channels. (a) the ten primary subtypes of Ca_v_ channels are classified into three groups-Ca_v_1, Ca_v_2, and Ca_v_3-based on the sequence similarities of the α1 subunits. The Ca_v_1 and Ca_v_2 channels belong to high-voltage activated (HVA) channels, while Ca_v_3 members are low-voltage activated (LVA) channels. Also shown are the genes that encode the α1 subunits and the current type characterized by electrophysiological experiments. The representative drugs of each isoform are listed. (b) chemical structures of small molecule drugs whose MOAs have been determined by high-resolution structures. Please refer to table 1 for PDB codes and additional details. (c) the HVA channels consist of at least three subunits: the transmembrane α1 subunit, the extracellular α2δ subunit, and the cytosolic β subunit. The α1 subunit comprises four homologous repeats (I-IV), each containing six helices (S1-S6). The S1-S4 helices of each repeat constitute the voltage-sensing domain (VSD), and four sets of S5 and S6 helices form the ion-conducting pore domain (PD). The S4 segments, carrying repetitive positively charged residues, are highlighted as yellow cylinders. The auxiliary α2δ subunit is composed of two polypeptides (α2 and δ) associated by an inter-subunit disulphide bond. The glycosyl phosphatidylinositol (GPI) group at the C-terminus of δ anchors it to the outer leaflet of the cell membrane. The α2δ subunit contains one von willebrand factor-A (VWA) domain and four cache domains. The two conserved domains of the β subunit, *src* homology 3 (SH3) and a guanylate kinase­-like domain (GK), are also shown. GK interacts with the α1-interacting domain (AID) motif located at the intracellular loop between repeat I and II.

Ca_v_ channels constitute a group of integral membrane proteins that facilitate the selective influx of Ca^2+^, a second messenger involved in numerous cellular events, into the cytosol upon membrane depolarization. These proteins exhibit diverse tissue distributions and play pivotal roles in a wide array of physiological processes, including muscle contraction, neurotransmitter release, hormone secretion, and cell death [1,2]. The history of Ca_v_ channel discovery has been comprehensively reviewed by Tsien, Barrett and Dolphin [3,4]. In mammals, the ten primary subtypes of Ca_v_ channels are classified into three subfamilies, Ca_v_1 (Ca_v_1.1-Ca_v_1.4), Ca_v_2 (Ca_v_2.1-Ca_v_2.3) and Ca_v_3 (Ca_v_3.1-Ca_v_3.3), based on sequence similarities of the α1 subunit (Figure 1a) [5–10]. As implied by the family name, the open probability of Ca_v_ channels is regulated by membrane potential. Ca_v_1 and Ca_v_2 are recognized as high-voltage activated Ca^2+^ (HVA) channels, given their activation at more depolarized membrane potential [1]. In contrast, Ca_v_3 channels form a subset of low-voltage activated Ca^2+^ channels (LVA) that can be activated at depolarization only slightly above the resting membrane potential and, in some cases, may even require conditioning hyperpolarization for priming [11,12].

Dysfunction of Ca_v_ channels is implicated in a range of disorders, including cardiac arrhythmias, primary aldosteronism, ataxia, migraine, cognitive anomalies, and autism. The physiology, pathology, and pharmacology of Ca_v_ channels have been extensively documented by Zamponi, Dolphin, *et al* [2,13–15]. For instance, small molecule drugs, e.g. 1,4-dihydropyridines (DHP, e.g. nifedipine), benzothiazepines (e.g. diltiazem) and phenylalkylamines (e.g. verapamil), that inhibit the cardiovascular L-type Ca_v_ channels (LTCCs), have been widely used in clinical practice for decades to treat hypertension and cardiac arrhythmias (Figure 1a,b) [16,17]. Simultaneously, peptide toxins from spiders, snakes, and marine creatures, which selectively bind to specific regions of ion channels, have historically been employed to investigate the subtype-specific inhibitory effects [18,19]. Achieving a precise understanding of the modes of actions (MOAs) of Ca_v_-targeting drugs requires the structural elucidation of drug-bound channels, a daunting challenge that was finally overcome thanks to the resolution revolution of single particle cryo-electron microscopy (cryo-EM).

The pore-forming α1 subunits of Ca_v_ channels exhibit nearly identical topologies, with approximately 2000 residues arranged into four homologous repeats (designated I-IV), each containing six transmembrane helices (S1-S6) (Figure 1c). The S5 and S6 segments from all four repeats collectively enclose the central pore domain (PD), forming a specific pathway for the selective passage of Ca^2+^ ions across the cell membrane. Meanwhile, the S1-S4 segments within each repeat constitute the voltage-sensing domain (VSD), where the S4 segment carries repetitive positively charged residues (arginine or lysine) as gating charges. The four sets of VSDs encircle PD in a domain-swapped manner, cooperatively coupling the fluctuations in membrane potential to the pore gating of ion conduction [5,20–23].

The core α1 subunit is self-sufficient for the autonomous function of Ca_v_3 channels, whereas the Ca_v_1 and Ca_v_2 subfamilies necessitate auxiliary extracellular α2δ and intracellular β subunits for proper membrane trafficking and physiological functions (Figure 1c) [11,24–26]. The Ca_v_1.1 channels, specialized for skeletal muscle, also associate with a transmembrane γ subunit sharing the same folding pattern with claudins [27,28]. Within the Ca_v_1 and Ca_v_2 subfamilies, the α1 subunits interact with the α2δ subunits through the extracellular segments, while interacting with the β subunits on the cytosolic side (Figure 1c). Four mammalian α2δ genes, namely *CACNA2D1 to D4*, encode the extracellular subunits α2δ-1 to −4, respectively [29]. These gene products are initially the preproteins for the α2δ subunits, which will undergo post-translational proteolysis into α2 and δ proteins as the mature forms. Nonetheless, these two proteins remain interconnected due to inter-subunit disulfide bond formation before proteolytic cleavage [30–33]. The mature α2δ subunits are highly glycosylated proteins that aid in trafficking the α1 subunits to the cell membrane, enhancing channel expression, and modulating channel properties [26]. In addition, four subtypes of β subunits, β1-β4 with multiple splice isoforms, contribute to channel trafficking, modulate gating properties, and interact with intracellular signaling molecules [24,34–38]. The specific assortment of subunit constituents varies depending on the particular type of Ca_v_ channel and the specific tissue or cell type in which it is located.

Obtaining accurate 3D structures for Ca_v_ channels lays the foundation for unveiling the working mechanisms and advancing drug discovery endeavors. The first cryo-EM structure of Ca_v_ channels was reported by our group in 2015 [28]. Following that, various subtypes of Ca_v_ channels alone and in complex with small molecule and peptide ligands have been extensively characterized (Table 1). This review will provide a comprehensive overview of recent advancements in the structural architecture and pharmacology of Ca_v_ channels, aiming to facilitate future physiological investigations and the development of innovative drugs.

**Table 1.** Published cryo-EM structures of Ca_v_ channels.

Protein	Species	Ligand	Resolution (Å)	PDB code	Refs
Ca_v_1.1	*rabbit*	None	4.2	3JBR	[28]
	*rabbit*	None	3.6	5GJV	[27]
			3.9	5GJW	
	*rabbit*	Nifedipine	2.9	6JP5	[39]
		(*S*)-(-)-Bay K8644	2.7	6JP8	
		Verapamil	2.6	6JPA	
		Diltiazem	2.9	6JPB	
	*rabbit*	(*S*)-(−)-Bay K8644	3.0	7JPK	[40]
			3.4	7JPL	
			3.4	7JPV	
		(*R*)-(+)-Bay K8644	3.2	7JPW	
		Levamlodipine	2.9	7JPX	
	*rabbit*	Amiodarone	2.8	8E56	[41]
		Amiodarone & MNI-1	2.8	8E57	
			3.0	8E58	
Ca_v_1.2	*human*	Gabapentin	3.1	8FD7	[42]
	*human* *rabbit*	L-leucine	3.3	8EOG	[43]
	*human*	None	2.9	8WE6	[44]
		Amiodarone & Sofosbuvir	3.3	8FHS	
		Calciseptine	3.2	8WE7	
		Pinaverium bromide	3.2	8WEA	
		Levamlodipine & Calciseptine	2.9	8WE8	
Ca_v_1.2-EMC	*human* *rabbit*	None	3.4	8EOI	[43]
Ca_v_1.3	*human*	None	3.0	7UHG	[45]
		Cinnarizine	3.1	7UHF	
	*human*	Amiodarone	3.1	8E59	[41]
		Sofosbuvir	3.3	8E5A	
		Amiodarone & Sofosbuvir	3.3	8E5B	
Ca_v_2.2	*human*	None	3.1	7MIY	[46]
		Ziconotide	3.0	7MIX	
	*human*	None	2.8	7VFS	[47]
		Ziconotide	3.0	7VFU	
		PD173212	3.0	7VFV	
		Ca_v_2.2 blocker 1	3.3	7VFW	
Ca_v_2.3	*human*	None	3.1	8EPL	[48]
			3.1	7XLQ	[49]
Ca_v_2.3 mutant	*human*	None	3.1	8EPM	[48]
Ca_v_3.1	*human*	None	3.3	6KZO	[50]
		Z944	3.1	6KZP	
Ca_v_3.3	*human*	None	3.3	7WLI	[51]
		Mibefradil	3.9	7WLJ	
		Pimozide	3.6	7WLL	
		Otilonium Bromide	3.6	7WLK	
α2δ-1	*human*	Mirogabalin	3.2	8IF3	[52]

## Structural architecture of Ca_v_ channels

Ca_v_ channels were first purified from the T-tubule membranes of rabbit skeletal muscle and later from the cardiac and brain membranes [53–58]. The endogenously purified rabbit Ca_v_1.1 channel has historically served as the prototype for the structural analysis of working and drug modulation mechanisms [27,28,39,40]. More recently, the establishment of a heterologous expression system for Ca_v_ channels has enabled the generation of sufficient samples encompassing diverse subtypes, facilitating the structural elucidation of a broad range of Ca_v_ channels [44]. In this section, we will delve into a comprehensive review of the precise structural architectures of representative members within each Ca_v_ subfamily.

### Subunit architecture of Ca_v_ channels

**Figure 2.**
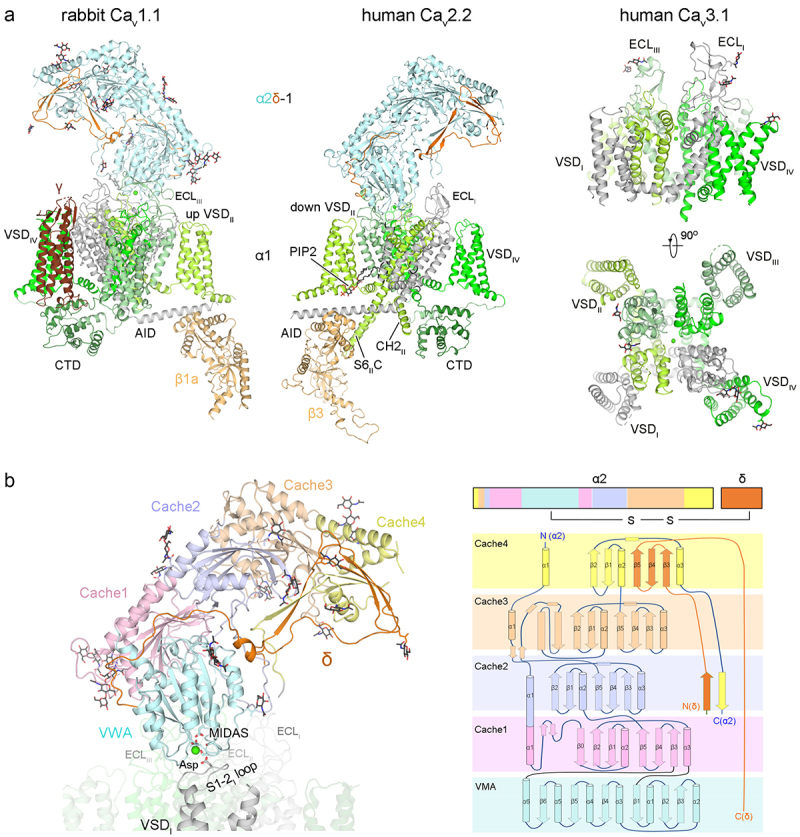
The subunit architecture of Ca_v_ channels. (a) the overall structures of rabbit Ca_v_1.1 (PDB: 5GJV), human Ca_v_2.2 (PDB: 7MIY) and human Ca_v_3.1 (PDB: 6KZO). All structures are domain-colored. In Ca_v_2.2, the β3 subunit is sandwiched between AID and the S6_II_ cytosolic segment (S6_II_C). The Ca_v_2-specifc cytosolic helix in repeat II (CH2_II_) is also labeled. The lipid molecule, phosphatidylinositol 4,5-bisphosphate (PIP2, black stick), is coordinated with the interface between down VSD_II_ and PD. CTD, C-terminal domain (forest cartoon). ECL, extracellular loop. (b) structural topology of the α2δ-1 subunit. The VWA domain and four cache domains (domain-colored) are intertwined in the primary sequence. The α1 subunit interacts with VWA (palecyan), Cache1 (light pink) and the intervening loop of the Cache2 domain (light blue) of the α2δ-1 subunit. The interface between VWA and α1 subunit involves the coordination of a Ca^2+^ ion (green sphere), facilitated by the metal ion-dependent adhesion site (MIDAS) site and the Asp residue on the S1–2 loop of repeat I.

The endogenously purified rabbit Ca_v_1.1 and recombinantly expressed Ca_v_2.2 channels both comprise a transmembrane α1 subunit, along with auxiliary α2δ-1 and β subunits. The rabbit Ca_v_1.1 was also recognized for its interaction with the γ subunit (Figure 2a, left). Examining the overall structures of representative members within the three Ca_v_ subfamilies provides detailed insight into their subunit architectures (Figure 2a).

The extracellular α2δ-1 subunit comprises a single von Willebrand factor-A (VWA) domain and four tandem Cache domains (Cache1 to 4), which are intertwined in the primary sequence (Figure 2b) [27,28]. Despite undergoing post-translational proteolysis, the α2δ-1 subunit remains covalently tethered through the disulfide bond between VWA domain and δ subunit. As observed in the structures, the VWA, Cache1, and the intervening loop of Cache2 domains are prominently engaged in interactions with the α1 subunit, particularly with the extracellular loops of repeats I to III (ECL_I_ to ECL_III_) and the intervening loop between S1 and S2 of repeat I (S1–2_I_ loop) (Figure 2a,b). Furthermore, the interface between VWA and α1 subunit involves the coordination of a Ca^2+^ ion, facilitated by the metal ion-dependent adhesion site (MIDAS) motif and the Asp residue on the S1–2_I_ loop of the α1 subunit.

The cytosolic β subunits have been identified to interact with the α1-interacting domain (AID), an 18-aa motif at the proximal region of the intracellular loop between domain I and II [59]. All β subunits feature a conserved *src* homology 3 (SH3) and a guanylate kinase­-like domain (GK), where the AID motif binds to the deep groove on the GK domain (Figure 2a). Besides the well-characterized interface, the β3 subunit was recognized for its additional interactions with the extended cytosolic segments of S6_II_ (S6_II_C) in the Ca_v_2.2 structure (Figure 2a, middle) [46].

In the Ca_v_1.1 structure, the γ subunit, which shares the same fold as claudins, interacts with VSD_IV_ through both transmembrane segments and cytosolic loops. The physical contacts between the γ subunit and the α1 subunit may potentially impact the conformational alterations of VSD_IV_ during voltage-dependent activation or inactivation. The interactions provide the molecular basis for the diverse modulation effects, including antagonistic influences, exerted by the γ subunit on channel properties (Figure 2a, left) [60,61].

### Diverse PD conformations in the structures of Ca_v_ channels

Four sets of transmembrane S5 and S6 along with the supporting helices P1 and P2 collectively form the central PD, serving as the high-throughput and highly selective pathway for the influx of Ca^2+^ ions. Inquiries into the precise mechanisms by which Ca_v_ channels specifically bind to Ca^2+^ ions and swiftly release them have held substantial importance for decades [21,62–64]. The high-resolution 3D structures provide the opportunity to reassess previous biophysical measurements, advancing our understanding of the selectivity and permeation of Ca_v_ channels.

**Figure 3.**
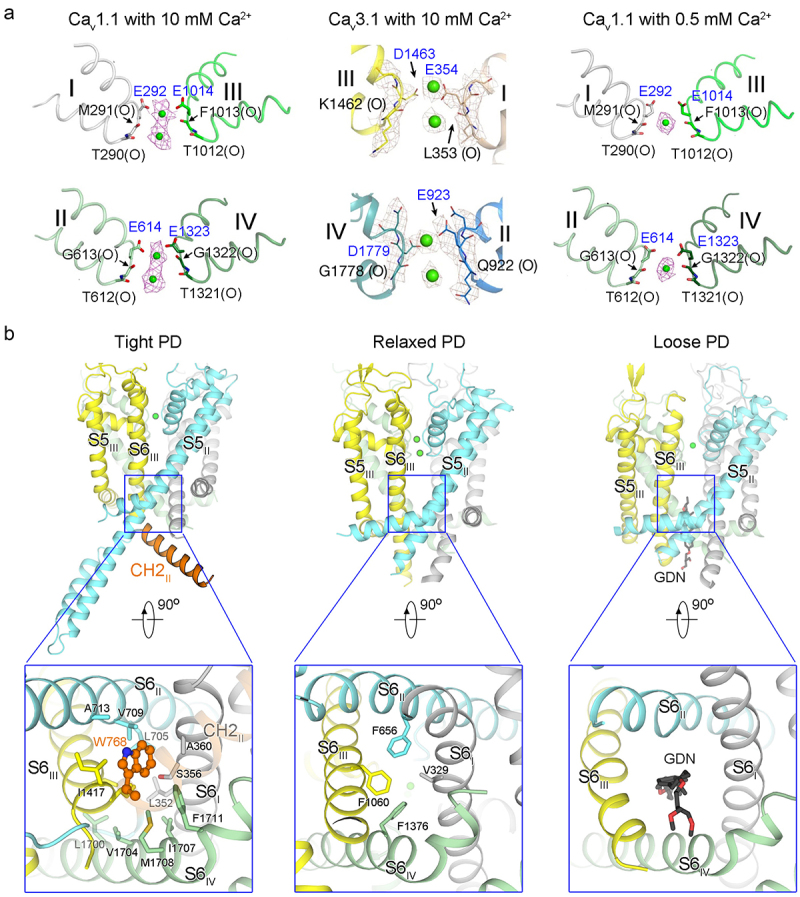
The structures of PD. (a) the putative Ca^2+^ coordination in SF vestibule. Ca^2+^ ions are coordinated by the EEEE residues for Ca_v_1 and Ca_v_2 channels, contrasting with EEDD residues in Ca_v_3 channels. The distinct features of Ca^2+^ ion densities (purple and brown meshes) are observed at concentrations of 0.5 and 10 mM, respectively. (b) the resolved structures of PD can be classified into three conformations, tight PD (represented by Ca_v_2.2, PDB: 7MIY), relaxed PD (represented by Ca_v_1.1, PDB: 5GJV), and loose PD (represented by Ca_v_1.2, PDB: 8WEA). The variations include the volume of the central cavity, the composition and size of the intracellular gate, and the contour of SF. The α1 structures are domain colored (grey for repeat I, cyan for repeat II, yellow for repeat III, and palegreen for repeat IV). The Ca_v_2-specific CH2_II_ segment is shown as orange cartoon, with the conserved Trp residue shown as orange ball-and-stick. The hydrophobic residues constituting intracellular gate are depicted as sticks. The detergent molecule, glyco-diosgenin (GDN), is nestle into the expanded intracellular gate, as seen in the loose PD. Panel a was adapted from our published papers [27,50] with minor modifications.

In the resolved structures, the EEEE/EEDD side chains protrude into the pore lumen, constituting the intrachannel binding site for Ca^2+^ ion (Figure 3a). The EM densities within the selectivity filter (SF) vestibule can be deconvoluted as two closely spaced Ca^2+^ ions: one situated directly at the EEEE/EEDD locus, and the other positioned in proximity to the carbonyl groups of the − 1 residues at a Ca^2+^ concentration of 10 mM (Figure 3a, left and middle). Moreover, the EEEE locus is coordinated with a single Ca^2+^ ion in the presence of 0.5 mM Ca^2+^ (Figure 3a, right). It was postulated that Ca_v_ channel comprised an intrapore Ca^2+^ binding site of low affinity (K_1/2_ ~10 mM), and an external binding site of high affinity (K_1/2_ ~0.3 μM) [65]. The presence of intrapore Ca^2+^ density at sub-millimolar Ca^2+^ concentrations supersedes the initial model of the low-affinity binding site within pore. Nonetheless, mutational studies have failed to reveal an additional high-affinity Ca_v_ binding site other than the one formed by EEEE residues [66,67]. Structural and mutational analyses suggest that the EEEE motif tightly binds a single Ca^2+^ to impede Na^+^ influx at low concentrations, while undergoing spatial rearrangements to permit Ca^2+^ influx as concentration increases [68].

Additionally, the structural gallery of Ca_v_ channels offers a glimpse into diverse conformations for the inactivated states. We propose classifying these states as “tight,” “relaxed,” and “loose” conformations to differentiate the structural variations within PD of the inactivated Ca_v_ channels (Figure 3b). The “tight” PD, evident in the structures of Ca_v_2.2 and Ca_v_2.3 exhibiting voltage-dependent closed-state inactivation (CSI) [69], features a smaller central cavity volume with only a minor fenestration (Figure 3b, left). The intracellular gate is notably thick and tightened, facilitated by the presence of a Ca_v_2-specific cytosolic helix at the II-III linker (designated as CH2_II_) [46–48]. Supporting the structural analysis of CH2_II_’s role in stabilizing the inactivated state, deletion of the CH2_II_ helix or mutation of the conserved Trp residue both exerted a substantial impact on channel inactivation, shifting the steady-state inactivation curves toward less negative voltages. Significantly, these mutations eliminated cumulative inactivation during action potential trains, indicating the crucial role of the CH2_II_ segment as a structural determinant for the CSI of Ca_v_2 channels [47]. The slightly “relaxed” PD, observed in most Ca_v_1 and Ca_v_3 channel structures, displays four fenestrations and a gate radius of approximately 1 Å (Figure 3b, middle). In these two types of conformations, the structure of SF remains relatively consistent. The “loose” PD configuration was recently identified in the structure of Ca_v_1.2 in the presence of an atypical calcium antagonist [44]. In this structure, the SF became dilated, and the gate, while not permeable, was sufficiently expanded to accommodate a detergent molecule (Figure 3b, right).

### The structural arrangement of VSD

It was suggested that the conformational changes in VSDs in response to membrane depolarization were initially attributed to the movement of gating charge residues on S4 segments across the membrane electric field [23,70,71]. As described in the sliding helix model, the transfer of gating charge residues through the hydrophobic constriction site is facilitated by the transient formation of ion pairs with countercharged residues on the opposite helices [71,72]. As a result, the gating charge residues are thought to “slide” through the lipid bilayer, facilitating the conformational changes in the VSDs that ultimately lead to the opening or closing of the ion channel pore.

**Figure 4.**
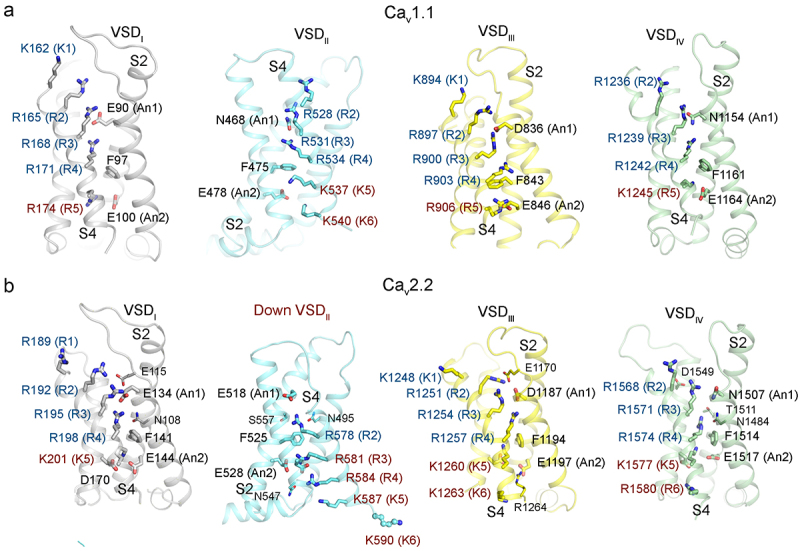
The structures of VSDs. (a) the depolarized conformations of the four VSDs, as seen in the Ca_v_1.1 structure (PDB: 5GJV). The gating charge residues on S4, An1 and An2 (conserved acidic or polar residues on S2), as well as the occluding Phe, are shown as sticks. The gating charge residues above and below the occluding Phe are labelled cyan and brown, respectively. In the depolarized conformation, R1-R4 are positioned above the occluding Phe. (b) the structure of the four VSDs in human Ca_v_2.2 (PDB: 7MIX). VSD_II_ (brown labeled) is in a down/deactivated conformation, while the other three VSDs remain in the depolarized state. In the down VSD_II_, the gating charge residues R3-K6 are below the occluding Phe (brown labeled), with only R2 above it (cyan labeled). K6 of VSD_II_ is on the opposite side to the other four gating charge residues and projects into the cytosol. Panel b was adapted from our published paper [46] with minor modifications.

The resolved structures of VSDs can be categorized into two classes. In the structures of Ca_v_1.1, Ca_v_1.3, and Ca_v_3 channels, all four VSDs are characterized as depolarized or up state. As depicted in the structure of Ca_v_1.1, the gating charge residues R1-R4 within the four VSDs are positioned above the conserved occluding Phe in the charge transfer center. Meanwhile, R5 and R6 are positioned below (Figure 4a). In contrast, the structures of Ca_v_2.2, Ca_v_2.3 and Ca_v_1.2 in the apo state reveal a down/deactivated state of VSD_II_, with the other three VSDs (VSD_I_, VSD_III_, and VSD_IV_) remaining in the depolarized state (Figure 4b). In the down VSD_II_, the entire S4 segment transforms into a 3_10_ helix, placing the gating charge residues on the same side. As shown in the structure of Ca_v_2.2, only R2 is situated above the occluding Phe, while R3-K6 are situated below it. Upon superimposing the four VSDs, S1-S3 are relatively well aligned, whereas S4_II_ shifts significantly downward compared to the other S4 segments.

To identify the determinants governing the conformational transition of VSD_II_, we suggest comprehensive investigations that include constructing Ca_v_1 and Ca_v_2 chimeras and performing systematic mutagenesis analysis. The endeavors will significantly enhance our comprehension of the electromechanical coupling mechanism.

## The molecular basis for Ca_v_ channels modulated by endogenous components

The activity of Ca_v_ channels is subject to modulations by a variety of endogenous components, such as calmodulin [73], G proteins [74], lipids [75], and synaptic associated proteins [76]. Recent breakthroughs in elucidating the specific interactions involving calmodulin [77,78], components of muscle excitation-contraction coupling (STAC and junctophilin) [79,80], phosphatidylinositol 4,5-bisphosphate (PIP2) [46,48], as well as chaperones in the assembly of Ca_v_ channels [81], have significantly deepened our understanding of Ca_v_ channel modulations.

**Figure 5.**
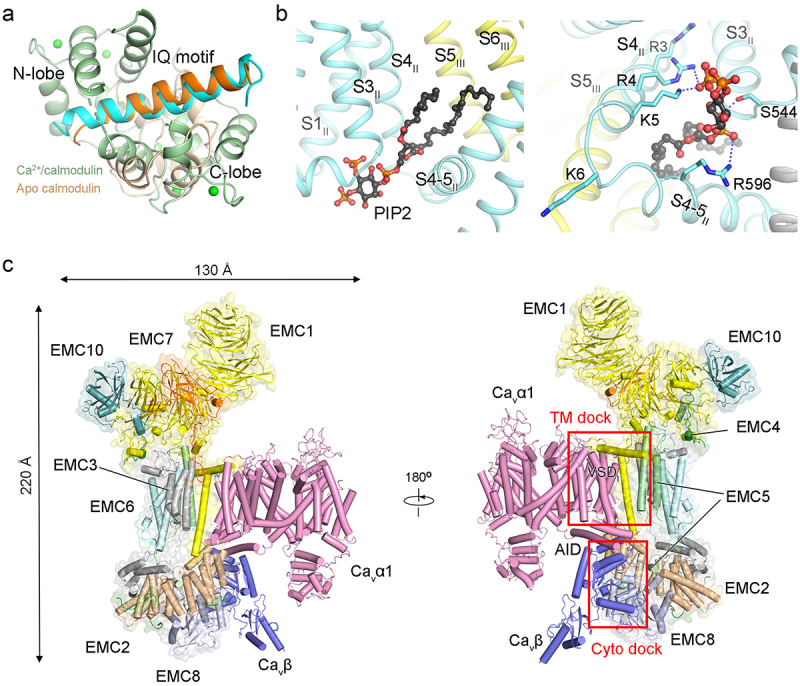
Structural basis for Ca_v_ channels modulated by endogenous components. (a) different binding modes of the Ca_v_ IQ motif (cyan and orange cartoon) with Ca^2+^/calmodulin (palegreen, PDB: 2BE6) or with apo calmodulin (wheat, PDB: 6CTB). Green spheres indicate Ca^2+^ ions. (b) binding details of PIP_2_ in the Ca_v_2.2 structure (PDB: 7MIY). *Left*: the PIP2 molecule is coordinated at the interface between down VSD_II_ and PD. *right*: the PIP2 molecule is coordinated with the polar residues in Ca_v_2.2. Hydrogen bond interactions are shown as blue dashes. (c) structure of the EMC chaperone-Ca_v_ assembly intermediate. The EMC – Ca_v_ is an approximately 0.6 MDa complex with dimensions of about 220 Å normal to the membrane plane and around 100 Å × 130 Å parallel to the membrane plane (PDB: 8EOI). EMC1–8 and 10, and the α1 and β3 subunits of Ca_v_1.2 are shown. The EMC extensively interacts with the α1 and β3 at the transmembrane (TM dock) and cytosolic regions (cyto dock).

Calmodulin functions as the Ca^2+^ sensor for calcium-dependent inactivation (CDI) of Ca_v_ channels [82]. We ever incubated human Ca_v_3.1 protein with calmodulin, but the final cryo-EM map revealed the absence of calmodulin and a large portion of C-terminal domain (CTD) [50]. Alternatively, insights into calmodulin-Ca_v_ interactions were gained through X-ray and NMR structural analyses using only isoleucine-glutamine (IQ) motif of Ca_v_. The IQ motif interacts with both N-lobe and C-lobe of Ca^2+^/calmodulin, with C-lobe showing higher affinity [77]. A recent NMR structure of Ca^2+^-free calmodulin (apo calmodulin) revealed that the IQ peptide interacted with the residues in C-lobe and adopted an orientation opposite to that in the structure with Ca^2+^/calmodulin (Figure 5a). The distinctive structural observations in Ca^2+^ or Ca^2+^-free calmodulin may provide insights into CDI regulated by the Ca^2+^ sensor protein [78].

Excitation-contraction coupling (ECC) in skeletal muscle necessitates functional and mechanical coordination between Ca_v_1.1 and the ryanodine receptor (RyR1) [83,84]. Recently, the Van Petegem group determined the structures of Ca_v_1.1 peptides bound to STAC2 (Ca_v_1.1 II-III loop) [80] and junctophilin 2 (Ca_v_1.1 CTD) [79], revealing the interactions of Ca_v_1.1 with the proposed auxiliary proteins involved in ECC.

PIP2 was initially observed to decelerate the “rundown” process and modify the voltage-dependence of Ca_v_2.1 channels, a finding later corroborated in Ca_v_2.2 channels [75,85,86]. In the structure of Ca_v_2.2 and Ca_v_2.3 channels, PIP2 is coordinated at the interface between the down VSD_II_ and PD (Figure 5b, left). The head group of PIP2 wedges into the cytosolic cavity within VSD_II_ through the interplay of S3 and S4, while the hydrophobic residues on S3 to S6 in repeat II, as well as S5 and S6 in repeat III, coordinate the tails of PIP2. Gating charge residues R4 and K5 interact with the 5-phosphate group of PIP2 (Figure 5b, right). Moreover, the Ca_v_2-specific CH2_II_ helix was noted to affect the coordination of PIP2 [48]. In the CH2_II_-deleted structure, the elevated shifts of S4–5_II_ and S5_II_ afford a more sharpened angle at the location where PIP2 was observed, rendering it incompatible for PIP2 binding.

The precise biogenesis processes play a pivotal role in ensuring the proper physiological functions of Ca_v_ channels ,[87]. In eukaryotes, the conserved large nine-protein complex, termed endoplasmic reticulum membrane protein complex (EMC), has been shown to aid in efficient membrane insertion, and serve as a holdase for partially assembled membrane proteins [88]. Various structural and mutagenesis studies have outlined the functional roles of the EMC and implied diverse interactions with client proteins [89][90][91]. The recent structure of Ca_v_ channel bound to EMC revealed extensive interactions at transmembrane and cytosolic docking sites (Figure 5c) [43]. Guided by the structural analysis, mutagenesis at the EMC-Ca_v_ interface, particularly at the Ca_v_β-Cyto dock, resulted in a substantial reduction in Ca_v_ channel currents. The structure-function analysis underscores the role of EMC as a channel holdase to facilitate the assembly and functional expression of Ca_v_ channels, thereby advancing our comprehension of the ion channel assembly intermediate.

## Structural pharmacology of Ca_v_ channels

In this section, we will present an overview of the recent advancements in the structural pharmacology of Ca_v_ channels. The structural gallery, revealing the diverse MOAs of small molecule drugs and peptide toxins, is expected to greatly accelerate the future drug discovery efforts targeting Ca_v_ channelopathies.

### Structural pharmacology of DHP drugs

DHP drugs, such as nifedipine, and amlodipine, have been extensively utilized in clinical settings for the effective management of hypertension. By inhibiting LTCCs in cardiovascular tissue cells, DHP drugs reduce peripheral vascular resistance and lower blood pressure [16,92,93].

**Figure 6.**
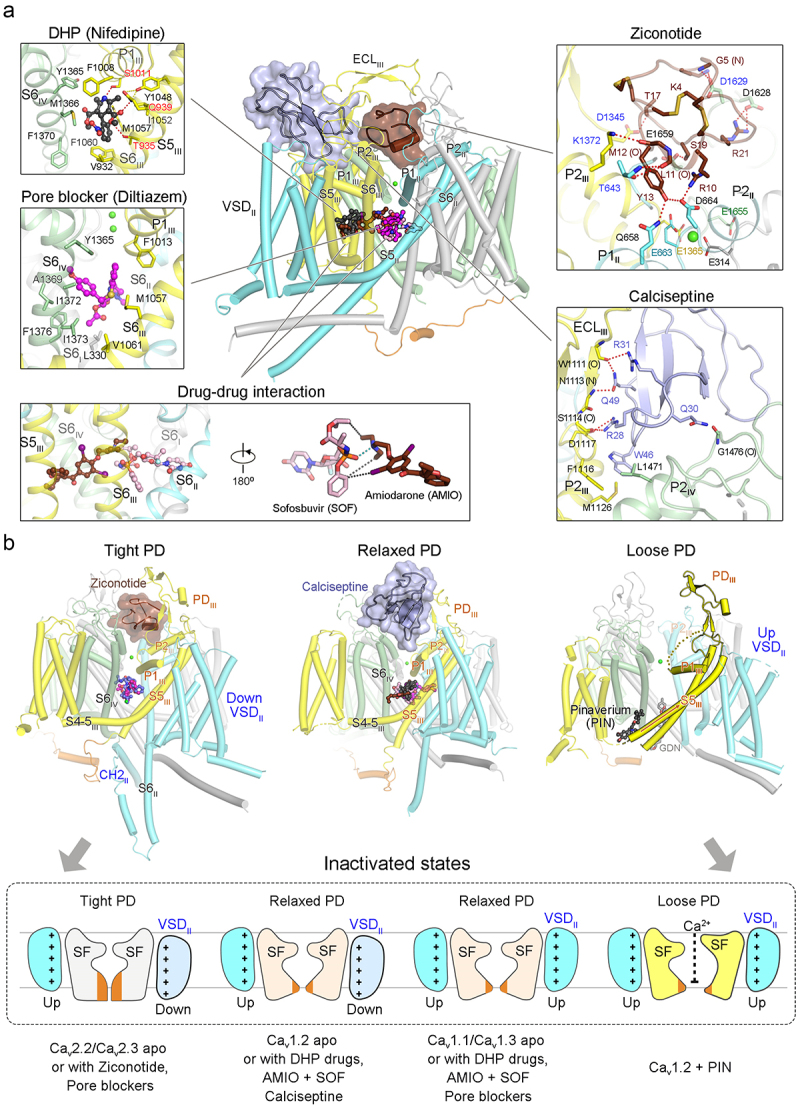
Overview of the structural pharmacology of Ca_v_ channels. (a) structural mapping of the drug binding sites in the Ca_v_ structures. Representative DHP drug (nifedipine, black ball-and-stick) and pore blocker (Diltiazem, magenta ball-and-stick) are shown. key interactions of representative drugs are summarized below. Hydrogen bonds are indicated by red dashes. *Nifedipine*: the N1 amine is hydrogen-bonded (H-bonded) to the hydroxyl group of Ser1011 on P1_III_, the two oxygen groups of the C3-ester are each H-bonded to Thr935 and Gln939 on S5_III_, the nitrophenyl ring is situated within a hydrophobic pocket formed by Val932 on S5_III_ as well as Met1057 and Phe1060 on S6_III_, the DHP backbone and the methyl groups are surrounded by Phe1008 on P1_III_ as well as Tyr1365 and Met1366 on S6_IV_ [39]; *Diltiazem*: interacts with the hydrophobic residues on S6_I_, P1_III_, S6_III_, and S6_IV_. Replacement of Tyr1365, Ala1369, and Ile1372 on S6_IV_ with non-LTCC residues was shown to significantly decrease the sensitivity to the drug [39]; *Drug-drug interaction*: AMIO (chocolate ball-and-stick) binds within the III-IV fenestration and mainly interacts with the hydrophobic residues on S6_III_ and S6_IV_. AMIO anchors the accommodation of SOF (pink ball-and-stick) into the cental cavity through hydrophobic contacts (black dashes) and a polar interaction (cyan dashes) between the phosphate group of SOF and the tertiary amine of AMIO [41]; *Ziconotide* (chocolate): Arg10 and Tyr13 of ziconotide are H-bonded to Asp664 on P2_II_, Ser19 is H-bonded to Glu1659 on P2_IV_, Thr17 is H-bonded with Asp1345 on ECL_III_, Arg21 and Lys4 interact with Asp1628 and Asp1629 on ECL_IV_, respectively. Four of the eight ziconotide-coordinating residues in Ca_v_2.2, Thr643, Asp1345, Lys1372 and Asp1629 (blue label), are not conserved in other Ca_v_ channels [46]; *Calciseptine* (light purple): Arg31 and Gln49 of CaS are H-bonded to Trp1111 (O) and Asn1113 (N) on ECL_III_, Arg28 is H-bonded to Ser1114 and Asp1117 on ECL_III_, Gln30 interacts with Gly1476 (O), Trp46 is surrounded by hydrophobic residues Phe1116 on ECL_III_, Met1126 on P2_III_, and Leu1471 on P2_IV_. Replacement of Ca_v_1.2-specific residues at the channel-toxin interface with their counterparts in Ca_v_1.1 or Ca_v_2.2 (D1117H, V1501Y, N1113K, and A1123W) exhibited reduced sensitivity to calciseptine, revealing the molecular basis for the subtype specificity [44]. (b) the diverse configurations of Ca_v_ channels in complex with various drugs and toxins. Pinaverium bromide (PIN) captures Ca_v_1.2 in an inactivated state with a loose PD. Upon PIN binding, the transverse S4–5_III_ helix combines with the S5_III_ segment to form a single straight helix (orange arrow). P2_III_ helix is missing in the cryo-EM map (yellow dashes). The dilated intracellular gate is fitted with a GDN molecule. Shown below is a schematic cartoon illustrating different conformations for the inactivated states. The drugs and toxins that prefer “tight,” “relaxed” and “loose” PD, as revealed by cryo-EM structures, are indicated. The binding details for ziconotide in panle A, and panel B were adapted from our published paper [44,46] with modifications.

The molecular mechanisms underlying the modulation of Ca_v_ channels by representative DHP drugs, including nifedipine, amlodipine, (*R*)-(+)-Bay K8644 and (*S*)-(-)-Bay K8644, have been elucidated using rabbit Ca_v_1.1 as the prototype [39,40]. The DHP drugs bind to the fenestration site enclosed by the pore-forming segments in Repeat III and IV (Figure 6a, DHP). Consequently, these drugs regulate Ca_v_ channels through an allosteric modulation mechanism instead of directly obstructing the ion-permeation path. The conserved N1 amine of the DHP core plays a crucial role by forming a hydrogen bond with surrounding residues [94,95]. Furthermore, the two oxygens of the C3-ester group, which appear to be crucial for antagonistic activities, are also H-bonded to the polar residues conserved in the Ca_v_1 subfamily [96,97].

Among DHP drugs, amlodipine demonstrates remarkable potency and a pH-dependent inhibitory impact [98]. The ethylamine side chain has been noted to extend toward the ion pore, interacting with a phospholipid molecule that transverses the central cavity. This unique MOA may distinguish the efficacy of amlodipine from that of other DHP drugs [40].

### Structural pharmacology of small molecule pore blockers

In addition to DHP drugs, a variety of small molecule drugs, exemplified by diltiazem and verapamil, are commonly used to manage cardiovascular conditions. Unlike DHP compounds, these drugs inhibit Ca_v_ activities by directly obstructing the ion-permeation path, classifying them as pore blockers. While the pore blockers accommodate into the proximal central cavity pockets, there are slight differences in the coordination details [39,45,47,51].

Diltiazem and verapamil serve as the prototypes for benzothiazepine and phenylalkylamine drugs, respectively, which are commonly used to treat hypertension and cardiac arrhythmias. In the resolved structures, diltiazem predominantly engages with numerous hydrophobic residues on S6_I_, P1_III_, S6_III_, and S6_IV_ (Figure 6a, Diltiazem) [39]. In contrast, verapamil potentially adopts two distinct binding modes [39]. In the first mode, verapamil is primarily coordinated by the hydrophobic residues on P1_II_, S6_II_ and S6_III_. The second mode, where verapamil interacts mainly with the hydrophobic residues on S6_IV_, aligns with the previous biophysical characterizations [99].

Cinnarizine functions as a nonselective Ca_v_ blocker employed in the treatment of motion sickness [100]. It was observed that cinnarizine binds within the central cavity of PD, with the styrene group interacting with the residues on SF vestibule [45]. The core scaffold, diphenylmethylpiperazine group, fits into the cavity formed by the hydrophobic residues on repeat II and III, thereby stabilizing the overall binding of the molecule. Beside steric blockage, local structural shifts resulting from cinnarizine binding induce an α→π transition of the helical turn constituting the intracellular gate, further constricting the ion-permeation pore.

The structural basis for pore blockage of LVA channels, T-type Ca_v_ channels, has also been elucidated [50,51]. Z944 exhibits characteristics of both a pore blocker and an allosteric antagonist [50]. In the high-resolution structure, Z944 interacts with the II-III fenestration rather than the typical DHP binding pocket at the III-IV fenestration [39]. The phenyl ring is positioned into the fenestration site, while the tert-butyl group is directed toward the ion pore, impeding the permeation of ions. Similar to the cinnarizine-bound structure, Z944 binding induces an α→π transition in S6_II_. A recent study has further unveiled analogous binding modes observed in Ca_v_3.3 channels complexed with mibefradil, pimozide, and otilonium bromide [51].

Two inhibitors, PD17312 and Ca_v_2.2 blocker 1, were observed to block Ca_v_2.2 channels through a dual mechanism involving both pore blockage and allosteric modulation [47]. Despite having distinct chemical structures, the two molecules occupy the same binding site (III-IV fenestration) within Ca_v_2.2. Unveiling the structural details of the III-IV fenestration site will facilitate the drug discovery efforts aimed at targeting Ca_v_2.2 for the treatment of chronic pain.

### Structural basis for the adverse interactions of sofosbuvir and amiodarone on LTCCs

Sofosbuvir, which targets NS5B polymerase of the hepatitis C virus, has achieved almost 100% cure rates for hepatitis C [101–104]. Nevertheless, co-administration with amiodarone, an antiarrhythmic drug that primarily inhibits diverse ion channels in heart, leads to a drug-drug interaction causing severe bradycardia [105–107]. Analysis of the cryo-EM structures of LTCCs in the presence of the two drugs uncovered a direct drug-drug interaction within the channels, shedding light on the concerning clinical observation [41].

Amiodarone binds to the III-IV fenestration of the LTCC through extensive hydrophobic interactions (Figure 6a, Drug-drug interaction). However, when administered alone, sofosbuvir (or its analogue MNI-1) is not present in the structure. The synergistic inhibition of Ca_v_ channels by amiodarone and sofosbuvir (or its analogue MNI-1) is clearly elucidated by the two drug-bound structures. Amiodarone anchors sofosbuvir/MNI-1 to the central cavity of LTCC, effectively obstructing the ion-conducting pathway. The drug-drug interaction is facilitated by the hydrophobic contacts and notably enhanced by a polar interaction between the phosphate group of sofosbuvir/MNI-1 and the tertiary amine of amiodarone. Observing the direct physical and pharmacodynamic interaction of amiodarone and sofosbuvir/MNI-1 on the scaffold of LTCCs underscores the clinically pertinent, potentially life-threatening nature of this drug-drug interaction [41].

### Gabapentinoids bind to the α2δ-1 subunit

Gabapentinoid drugs are widely used to treat epilepsy, post-herpetic neuralgia, diabetic neuropathy, fibromyalgia, and anxiety disorder. Unlike the above-mentioned MOAs, gabapentinoids are recognized for interacting with the α2δ subunit, specifically the α2δ-1 and α2δ-2 isoforms, thereby reducing the membrane expression of the neuronal Ca_v_ channels [108–113].

The molecular recognition of the gabapentinoid drugs, gabapentin and mirogabalin, by the α2δ-1 subunit have been investigated, but the structural observations are somewhat ambiguous [42,52]. Gabapentinoid drugs were supposed to occupy a pocket identical to the L-leucine binding site at the Cache1 domain (Figure 2B). No discernible conformational change was observed in the α2δ-1 subunit upon binding to gabapentin or mirogabalin. However, our group ever refrained from conclusively assigning densities in the same site to gabapentinoid (pregabalin) due to its similar size and shape to the endogenous L-leucine ligand [27]. It remains to be investigated how gabapentinoids regulate the function of neuronal Ca_v_ channels if there is no conformational alternation for the α2δ-1 subunit.

### Structural basis for Ca_v_ channels blocked by peptide toxins

Peptide toxins have historically been valuable tools for understanding the physiological functions of ion channels. Moreover, ongoing advancements in peptide chemistry, drug delivery, and formulation are making peptide toxins an increasingly attractive avenue for the development of ion channel drugs [114].

Ziconotide, derived from ω-conotoxin MVIIA, acts as a selective blocker for Ca_v_2.2 channels and received FDA approval in 2004 for the treatment of severe chronic pain [18,19,115]. Structural studies have unveiled the molecular underpinnings of the specific pore blockage of ziconotide on human Ca_v_2.2 [46,47]. Ziconotide is situated in the electronegative pocket surrounding the entrance to SF (Figure 6a, Ziconotide). To accommodate ziconotide, ECL_III_ must move upward together with the α2δ-1 subunit. The positively charged residues in ziconotide effectively neutralize the negatively charged pocket that attracts ions into the ion entrance, creating a spatial obstruction to prevent Ca^2+^ influx into the pore. Further sequence comparison revealed that half of the crucial residues required for ziconotide coordination were not conserved among Ca_v_ channels, thus elucidating the molecular basis for the selective pore blockade of ziconotide.

Calciseptine, a member of the three-finger toxin isolated from the venom of black mamba, consists of 60 amino acids and features four pairs of disulfide bonds. With the ability to selectively block Ca_v_1.2 and Ca_v_1.3 channels, calciseptine effectively inhibits the contraction of smooth and cardiac muscles [116–121]. In contrast to other VSD or PD-binding toxins, calciseptine is positioned on the PD shoulder and thoroughly interacts with the P2 pore helices and the ECLs in repeats III and IV (Figure 6a, Calciseptine) [44]. Mutation assays have highlighted the role of Ca_v_1.2-specific residues, mapped at the protein-toxin interface, in determining the subtype specificity. The unexpected binding mode of calciseptine provides a valuable template and induces novel concepts for the development of innovative antihypertensive medications.

### Pinaverium bromide traps Ca_v_1.2 in an inactivated state with a loose PD

Studies in structural pharmacology not only clarify the MOAs of various drugs, but also have the potential to capture additional functional states of Ca_v_ channels. However, most investigations have yielded the conformations similar to the apo state. For instance, the structures of Ca_v_.2.2/Ca_v_2.3 in the apo state or in complex with drugs often exhibit an inactivated state with a tight PD (Figure 6b, left). Additionally, the structures of most drug bound LTCCs show a relaxed PD, similar to the apo structure (Figure 6b, middle). Our group recently revealed that pinaverium bromide (PIN), an antispasmodic agent used to alleviate symptoms of irritable bowel syndrome, could trap Ca_v_1.2 in a conformation distinct to the apo state [44].

PIN potentially wedges into the corner enclosed by S4–5_III_, S6_III_ and S6_IV_. Upon binding to PIN, Ca_v_1.2 undergoes a substantial range of conformational changes, including a structural transition of VSD_II_ from a down to an up state, a significant reshaping of SF vestibule, and dilation of intracellular gate (Figure 6b, right, Loose PD). Despite the considerable expansion of the ion-permeation path, molecular dynamics simulations reveal that the PIN-bound state remains non-conductive for Ca^2+^ ions, thereby supporting the antagonistic activities of PIN for Ca_v_1.2.

## Conclusion

Ca_v_ channels are of great physiological importance across cardiovascular, neuronal, and endocrine systems, serving as primary targets for treating diseases such as hypertension, cardiac arrhythmia, chronic pain, and epilepsy. In this review, we have summarized the strides made and novel insights achieved through the structural analyses of different Ca_v_ subtypes. The structure gallery establishes the foundation for dissecting the physiological functions of Ca_v_ channels and underlying the electromechanical coupling mechanisms. Moreover, the elucidation of structures in complex with clinically relevant drugs and promising ligands offers detailed insights into the structural pharmacology of Ca_v_ channels. These studies advance our comprehension of the working mechanisms, and pave the way for prospective drug discovery against Ca_v_ channelopathies.

## Data Availability

Data sharing is not applicable to this article as no new data were created or analyzed in this study.
